# FOSTERING AGE-FRIENDLY INITIATIVES IN SMALL UNIVERSITIES: A CASE STUDY OF SOUTHERN OREGON UNIVERSITY

**DOI:** 10.1093/geroni/igae098.1683

**Published:** 2024-12-31

**Authors:** Noriko Toyokawa

**Affiliations:** Southern Oregon University, Ashland, Oregon, United States

## Abstract

Compared to their larger counterparts, small U.S. higher education institutions face distinct challenges in implementing Age-Friendly University (AFU) initiatives. This presentation spotlights the AFU initiatives that Southern Oregon University (SOU) implemented as the case of such a higher education institution. Despite its resource constraints, such as the lack of aging-centered academic programs and an on-campus aging institute, SOU exemplifies AFU principles by its faculty’s developing courses to involve retired individuals through community partnerships and cultivate meaningful intergenerational interactions. While discussions on lifespan development topics provided in those courses have produced positive learning outcomes, they have also unveiled areas for improvement. Older adults desire more direct involvement to share their wisdom and expertise with students. In response, incorporating age diversity as a valuable resource, SOU strategically plants to enhance existing academic programs by infusing innovation into courses and promoting mutual learning between generations. For instance, a course in Adult Development and Aging is being re-designed so that collaborative groups of students and older adults will utilize electronic design tools and artificial intelligence to craft a book conveying older adults’ messages for the next generation. This addresses the needs of older adults and enhances technology skills for both groups. After examining the university’s current effort in the AFU initiative, this presentation will delve into small institutions’ unique challenges in promoting AFU initiatives. Recommendations will be explored, emphasizing the vital role of adapting existing courses to foster sustainable lifelong learning across generations.

